# Precipitation Effects on Microbial Pollution in a River: Lag Structures and Seasonal Effect Modification

**DOI:** 10.1371/journal.pone.0098546

**Published:** 2014-05-29

**Authors:** Andreas Tornevi, Olof Bergstedt, Bertil Forsberg

**Affiliations:** 1 Occupational and Environmental Medicine, Department of Public Health and Clinical Medicine, Umeå University, Sweden; 2 Department of Civil and Environmental Engineering, Water Environment Technology, Chalmers University of Technology, Gothenburg, Sweden; Catalan Institute for Water Research (ICRA), Spain

## Abstract

**Background:**

The river Göta Älv is a source of freshwater for 0.7 million swedes. The river is subject to contamination from sewer systems discharge and runoff from agricultural lands. Climate models projects an increase in precipitation and heavy rainfall in this region. This study aimed to determine how daily rainfall causes variation in indicators of pathogen loads, to increase knowledge of variations in river water quality and discuss implications for risk management.

**Methods:**

Data covering 7 years of daily monitoring of river water turbidity and concentrations of E. coli, Clostridium and coliforms were obtained, and their short-term variations in relation with precipitation were analyzed with time series regression and non-linear distributed lag models. We studied how precipitation effects varied with season and compared different weather stations for predictive ability.

**Results:**

Generally, the lowest raw water quality occurs 2 days after rainfall, with poor raw water quality continuing for several more days. A rainfall event of >15 mm/24-h (local 95 percentile) was associated with a three-fold higher concentration of E. coli and 30% higher turbidity levels (lag 2). Rainfall was associated with exponential increases in concentrations of indicator bacteria while the effect on turbidity attenuated with very heavy rainfall. Clear associations were also observed between consecutive days of wet weather and decreased water quality. The precipitation effect on increased levels of indicator bacteria was significant in all seasons.

**Conclusions:**

Rainfall elevates microbial risks year-round in this river and freshwater source and acts as the main driver of varying water quality. Heavy rainfall appears to be a better predictor of fecal pollution than water turbidity. An increase of wet weather and extreme events with climate change will lower river water quality even more, indicating greater challenges for drinking water producers, and suggesting better control of sources of pollution.

## Introduction

Drinking water producers are responsible for providing safe drinking water. One challenge faced by producers is that water supplies, especially surface water sources, may experience temporal variations in water quality. Therefore, the raw water quality needs to be repeatedly tested to validate if the current water treatment technique is sufficient for producing safe and clean drinking water. A common indicator of water quality is turbidity, a measure of water cloudiness, which is relatively easy to quantify with optical devices and is regularly used as a first indicator of levels of microbial contamination. However, turbidity reflects the load of organic and inorganic particles, so additional water samples are needed for more precise analysis of organic contaminants. Density analyses of indicator bacteria, such as coliforms or Escherichia coli, may return a better estimate of levels of human pathogens. Because precise analyses of indicator bacteria are performed in laboratories, there is a delay between sampling and results being available, which is why turbidity monitoring and indicator bacteria samples often complement each other.

Several studies have shown relationships with prior weather events, especially wet weather, and raw water quality parameters [1]–[3]. Heavy rainfall has also been linked to the majority of observed drinking water-related outbreaks of gastrointestinal diseases in developed nations worldwide [4]–[7]. However, the reported outbreaks may only represent a fraction of the total impact; the proportion of gastrointestinal infections caused by drinking water is argued to be much higher [8].

Within Sweden, the southwest is one of the regions with the highest annual precipitation, with an average of around 1 m per year, and climate change is projected to increase annual precipitation and heavy rainfall events. The river Göta Älv runs through this region, serving as a freshwater supply for 0.7 million people. The river is at risk of pathogen contamination from multiple sources, including runoff from agricultural land and point sources such as sewer system discharges [3]. More frequent events of heavy rainfall may increase the risk of contamination, increasing the challenges of providing safe water drinking water [9], [10].

### Aim

With the use of time series analysis we aim to describe how daily rainfall influences raw water quality parameters in this important freshwater supply in Sweden. We focus on water quality parameters commonly used as indicators of pathogen contamination and study their long term variations and determine precipitation effects on short term variations. We analyze the distribution of lagged effects of precipitation and determine the extent to which there are seasonal effect modifications. Such results will increase knowledge of fluctuations in raw water quality, and provide a causal link behind health effects and the evidence base to develop guidance for risk evaluation of drinking water production.

## Materials and Methods

### Study area

**Figure 1:**
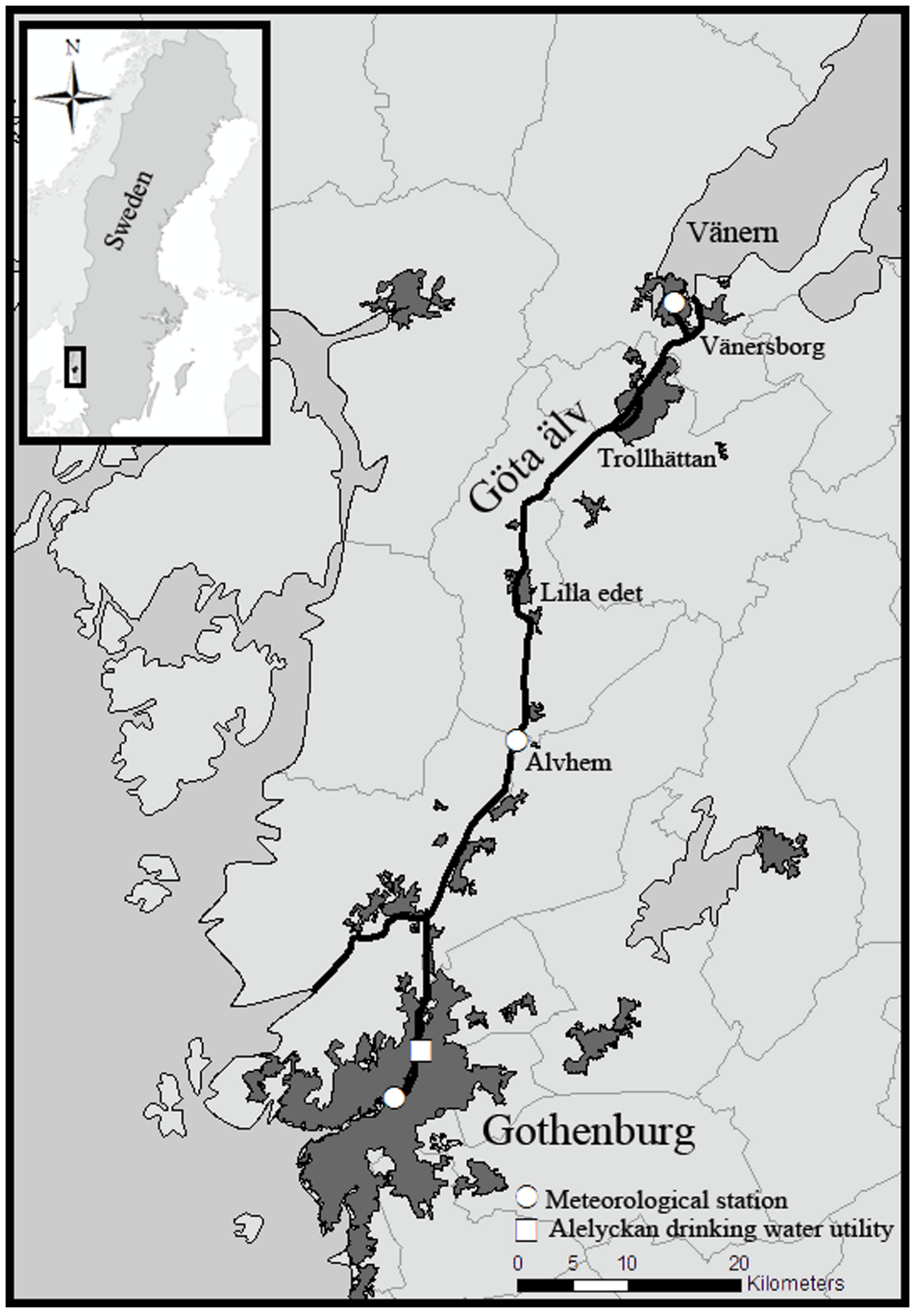
Map. Map of study area.

The river Göta Älv originates from Sweden's largest lake (Vänern) and runs 93 km to Kattegatt at the North Sea. Close to the sea, the river divides into a second branch (Nordre Älv), with Göta Älv continuing through the coastal city Gothenburg (latitude 57.708, longitude 11.975) (Figure 1). The City of Gothenburg is Sweden's second largest city with a population of about 500,000.

The main river arm has an average flow rate of 550 m^3^/s and varies around 200 to 1000 m^3^/s. Hydropower stations in the upper region (Vänersborg, Trollhättan and Lilla Edet) can cause rapid changes in stream flow. The flow time from Vänern to the sea varies from 1.5 to 5 days, with an average of about 3 days. The rivers total descent is about 44 meters, where the majority takes place at the hydropower stations.

Five drinking water utilities use the river as a freshwater supply, distributing water to around 700,000 people. Two of these utilities, Alelyckan and Lackarebäck, are distributing drinking water to the population in Gothenburg. Lackarebäck takes water from a lake reservoir (Delsjön) that under normal conditions is continually supplied with river water through a 9 km tunnel designed to maintain a constant water level in the lake. The river water intake at Alelyckan is located close to the utility and takes about 2 m^3^/s, with about half the volume for Alelyckan and the other half for the reservoir. This river water intake is closed when information suggest the river water is inadequate for drinking water production. To maintain drinking water production at Alelyckan, the water in the tunnel is then taken back and the tunnel transports freshwater in the other direction. Closures of the river water intake are determined by analysis of indicator bacteria and turbidity levels, or high conductivity caused by inflows of seawater. Intake closures can also be based on events of heavy rainfall or information on upstream events indicating elevated contamination risks, such as releases of untreated sewage water. The river is the recipient for eight wastewater treatment plants upstream of the intake at Alelyckan.

Despite the northern latitude of the study area, the climate is comparably very mild. The latitude provides distinct winter and summer seasons and daylight spans between 7–17 h/day. On average, February is the coldest month with daily mean temperatures a few degrees Celsius below zero. The river is usually not covered with ice during winter seasons. Precipitation is fairly constant throughout the seasons although the second half of the year generally experiences more and heavier rainfall events.

### Data

#### River water

**Figure 2:**
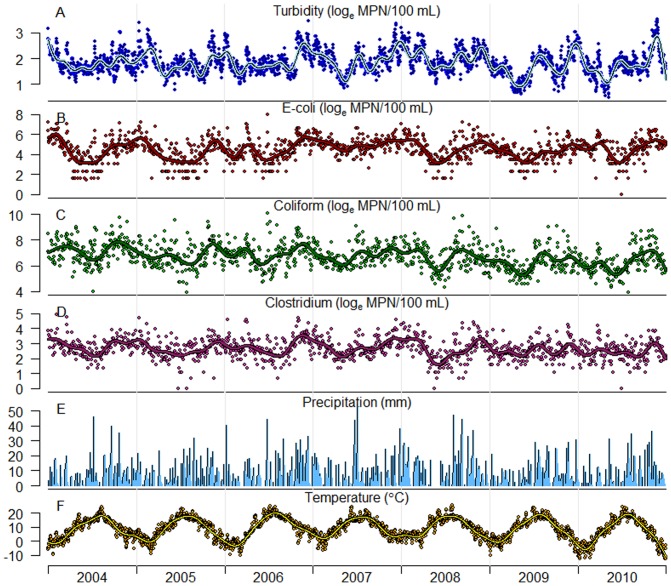
Data. Time series data for 2004 through 2010 for (from top) A: turbidity, B: E.coli, C: coliforms, D: Clostridium (river water intake Alelyckan), E: precipitation (Alvhem), and F: daily mean temperature (Gothenburg). A moving average is projected with a spline using 10 df/year for turbidity and 7 df/year for indicator bacteria and temperature. Data transformed by the natural logarithm (A–D).

Concentrations of the indicator bacteria Escherichia coli (E. coli), Clostridium perfringens and coliforms, together with water turbidity, are routinely monitored to indicate the quality of the river water. Seven stations along the river monitor quality parameters with varying frequencies. The municipal water department in Gothenburg, Department of Sustainable Waste and Water, provided laboratory analyses of concentrations of indicator bacteria, sampled outside the river water intake to Alelyckan during the time period 2004–2010. This location had the highest rate of sampling of indicator bacteria for laboratory analysis, three times weekly, although more frequent sampling may be performed, particularly when high concentrations are detected. Laboratory results of coliforms and E. coli were reported in units of Most Probable Number (MPN), water samples analyzed with Coliert-18/Quanti-Tray (ISO 9308-2:2012), enumerated after an incubation time of 18 hours at 35°C. Clostridium concentrations were reported in Colony Forming Unit (CFU), enumerated after incubation at 44°C for 21±3 hours (ISO/CD 6461-2 2002-12-20). These samples were not preheated with intention to kill vegetal cells. The analytic methods of enumeration of indicator bacteria have been the same during the study period. Turbidity has been continuously monitored at the river water intake and we obtained daily mean values in Formazin Nephelometric Unit (FNU) during the same period (2004–2010). Daily data on stream flow (m^3^/s) measured near Lilla Edet were also provided. The time series data of water quality parameters are displayed in Figure 2.

#### Weather

The Swedish Metrological and Hydrological Institute (SMHI) provided daily weather data. Daily precipitation data were available for three stations along the river: Gothenburg, (south station) the village of Alvhem situated about 30 km upstream from the water intake (middle station) (Figure 2), and the City of Vänersborg situated around 90 km upstream (north station), where also data on snow depth were available. Daily mean temperature data measured in Gothenburg (Figure 2) were also obtained.

### Statistical methods

The associations between daily precipitation and raw water quality were analyzed using time series regression. Raw water quality parameters were log-transformed (natural logarithm) and generalized additive regression models (GAM) were fitted [11]. We adjusted for long-term trends and seasonality patterns and analyzed how short-term effects of daily precipitation were distributed with Distributed Lag Non-linear Models (DLNM) [12], and unconstrained distributed lag models. We compared different weather stations for their predictive ability analyzed possible confounding factors such as change in upstream snow depth and temperature variations, and examined if weekday patterns were present. In addition we examined how consecutive wet or dry weather affect daily water quality. Finally, as the study area has distinct seasonality in temperature, daylight, ecology etc., we studied if short term effects of precipitations on raw water quality parameters vary with season and possible effect modifications due to river flow rate. This was done by only including specific time periods of data in regression analyses. Details on the statistical methods are described in a supplementary material (Text S1). The statistical software R (v 2.15.2) [13], together with MGCV [11] and DLNM [14] packages were used.

## Results

### Descriptive data and seasonal patterns

Daily mean values of river water turbidity ranged between 1.6 and 33.7 FNU with an overall mean of 7.0 FNU. The median value of E. coli was 96 MPN/100 mL and the highest concentration was enumerated to 2800 MPN/100 mL. Same statistics for coliforms and was 750 MPN/100 mL and 24000 MPN//100 mL. Although these maximum concentrations were detected in June, the best average river water quality occurred in spring and summer (April–July). In general, average water quality was lowest in the darker seasons, as for example maximum observation of turbidity in May was below the average value for November. Coliforms showed less elevated concentrations in December through March relatively the other water quality parameters, and also showed a long-term declining trend throughout the study period.

Most precipitation was recorded at the middle station with an average daily precipitation of 3.1 mm, or 7.0 mm if only including days with precipitation, resulting in an average annual precipitation of 1138 mm. The average annual precipitation in the other stations was 872 mm/year (north) and 970 mm/year (south). The maximum daily precipitation was recorded at the south station (59.7 mm), which also had the highest total count of days with any observed precipitation. Figure S1 illustrates seasonal patterns of weather and water parameters, along with all data observations. Descriptive data on river water parameters and weather observations are shown in Table 1, and in Table S1 monthly statistics about water parameters is displayed.

**Table 1:** Summary statistics of observations in river water and weather during 2004–2010.

	N	Missing	Mean	St.Dev	Minimum	25%:tile	Median	75%:tile	Maximum
Temperature °C (south)	2556	1	8,8	7,48	−12,7	3,4	9,1	15.0	25,4
Precipitation mm (middle)	2557	0	3,1	6,1	0	0	0	3,8	54,3
Precipitation mm (south)	2557	0	2,7	5,5	0	0	0,1	3	59,7
Precipitation mm (north)	2557	0	2,4	4,9	0	0	0	2,5	49,5
Stream flow m^3^/s (middle)	2555	2	566	216	148	416	571	730	1021
Coliforms MPN/100 mL	1156	1400	1374	1896	51	410	750	1600	24000
E. coli MPN/100 mL	1156	1400	156	200	1	41	96	190	2800
Clostridium CFU/100 mL	1054	1502	18,2	15,5	1	9	14	21	140
Turbidity FNU	2550	7	7,0	3,8	1,6	4,7	5,9	8,2	33,7

### Main effects of precipitation

Short-term variations in all raw water quality parameters were highly associated with prior precipitation regardless which weather stations provided data, although the middle station had the best predictive ability and the north station second best. An event of heavy precipitation decreased water quality over several days, with the effect peak in general two days later. Precipitation effects were significant over numerous lags, and the lag structure on turbidity appeared less peaky and to be affected a few days longer compared to indicator bacteria.

**Figure 3:**
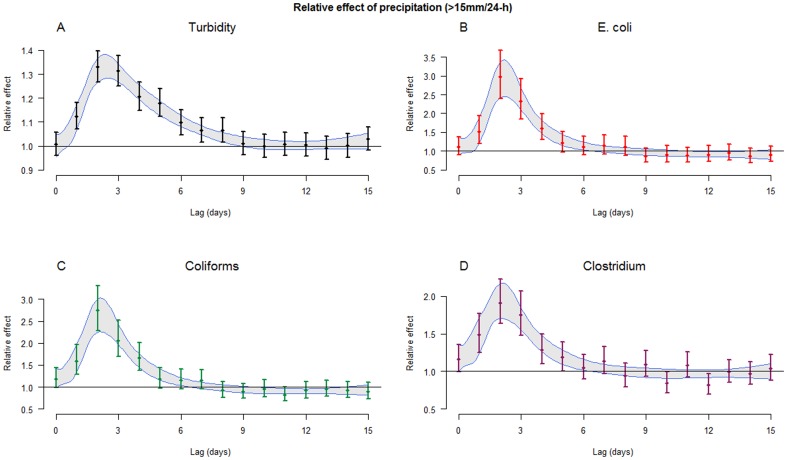
Effect of heavy precipitation across lags. Estimated relative effects of precipitation events (>15 mm/24-h) on river water quality parameters along 0 to 15 lag days: A: turbidity, B: E. Coli, C: coliforms, D: Clostridium perfringens. 95% confidence intervals are illustrated with bars (unconstrained distributed lag model) and with shaded area (DLNM model).

Precipitation events of 15 mm/24-h or more (n = 142) were associated with a three-fold increase in E. coli concentrations (+190%, 95% confidence interval (CI): 146–242%) two days later (lag 2) using a DLNM model. A similar model, with turbidity as outcome, estimated an average increase of 32% (CI: 27–37%) at lag 2. Figure 3 illustrates the relative effects on raw water quality parameters along lags 0 to 15, using both DLNMs and unconstrained distributed lag models.

**Figure 4:**
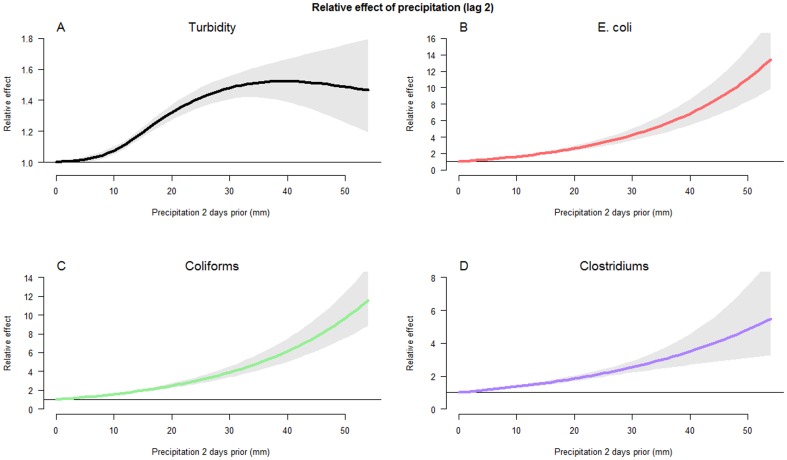
Precipitation effects lag 2. Relative effects of daily precipitation (0–54 mm) two days later on A: turbidity, B: E. Coli, C: coliforms, D: Clostridium perfringens. Gray areas represent 95% confidence intervals.

A DLNM model with precipitation as continuous predictor, and allowing for non-linear associations, fitted increased concentrations with increased amount of precipitation analyzing indicator bacteria, while the effect on turbidity attenuated with extreme rainfall. An event of 40 mm/24-h was estimated to increase E. coli concentrations two days later by 580% (CI: 443–754%), and an extreme event of 50 mm/24-h was associated with increased concentrations by 11 times (+1000%, CI: 730–1360%). The precipitation effects at lag 2 are illustrated in Figure 4, and the associations along all lags (0–15) with quantity of precipitation (0–54 mm) are illustrated in Figure S2.

**Figure 5:**
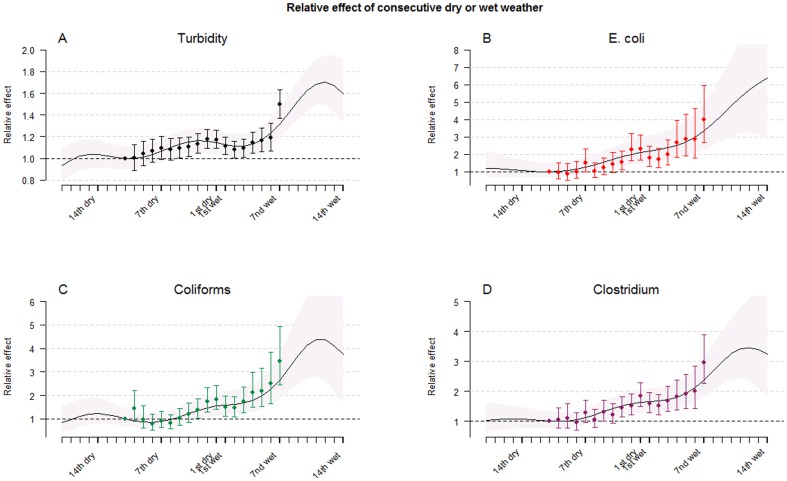
Effect of consecutive days with dry or wet weather. Relative effects (with 95% CIs) of consecutive wet or dry weather (middle station) on A: turbidity, B: E. Coli, C: coliforms, D: Clostridium perfringens. The reference is set to ten consecutive dry days using a continuous predictor (shaded), and at least ten dry days using a categorical predictor (bars). Seven wet days (bar) represent seven wet days or more.

Analyzing the effect of consecutive wet or dry weather days and raw water quality also exposed clear relationships. On average, consecutive wet weather for more than a week increased E. coli concentrations four-fold compared to one week of dry weather (+299%, CI: 168–492). As estimates for singular precipitation events, this precipitation predictor also indicated a lagged effect after wet weather days. Figure 5 displays the estimated effects of consecutive dry and wet weather on raw water quality parameters using a categorical predictor together with a smooth association using a penalized spline function, which both result in similar associations.

### Precipitation effect modifications

**Figure 6:**
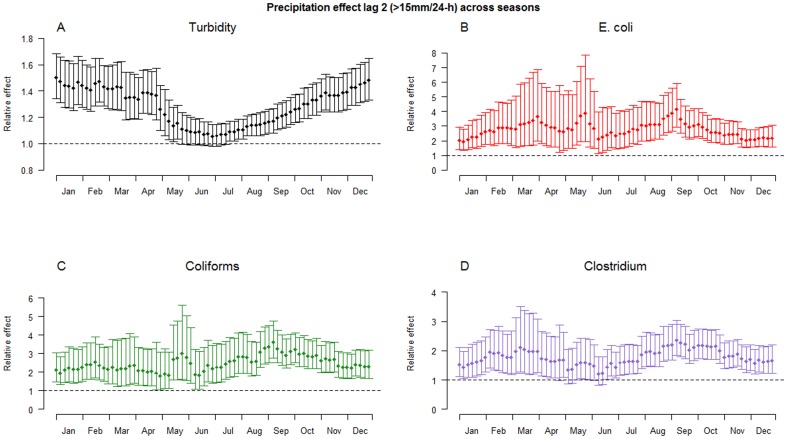
Effect modifications lag 2 across season. Relative effects of precipitation (>15 mm/24-h) 2 days later across seasons on A: turbidity, B: E. Coli, C: coliforms, D: Clostridium perfringens. Each estimate covers a range of 90 days: 45 days prior and 45 days following. Vertical bars represent 95% confidence intervals.

Analysis of seasonal effects indicated that precipitation influenced concentrations of indicator bacteria all year around and seasonal effect modifications were quite moderate. The effect of precipitation across the seasons on turbidity appeared to be modified more than for indicator bacteria, with the smallest relative effect during summer. Figure 6 illustrates the estimated effects two days after precipitation events (>15 mm/24-h) across seasons, and Figure S3 illustrates such events across lags 0–8 in a colored contour plot.

The extent to which short-term variations in raw water parameters were explained with prior precipitation varied over the year. The coefficient of determination (R^2^) for precipitation predictors logically indicated a similar seasonal pattern as the seasonal moving average of precipitation, but the variability differed between water quality parameters. Precipitation explained around 50 % of the variation in coliform concentrations in river water during fall, and decreased to around 20 % in winter and spring. When analyzing turbidity in summer periods the R^2^ values were low, especially when using precipitation data from the south station. R^2^ values of precipitation across season, from different weather stations, are displayed in Figure S4.

Analyzing precipitation effects under different stream flows exposed effect modifications in the expected direction; precipitation during lower flow rates spreads the effect over more lags together with a decreased peak effect, while during higher rates a more concentrated lag distribution with a higher effect peak was estimated. Figure S5 illustrates the lag structures on turbidity after rainfall events (>15 mm/24-h) in episodes separated by stream flows quartiles, where effect peaks ranged between ∼2 to 3.5 lag days.

### Model details and covariates

Estimates of precipitation effects presented were generated with observations from the middle station (Alvhem). The DLNM models were fit with natural cubic splines in lag space and used 6 df, with knots distributed at a log scale. DLNM models with continuous precipitation predictor were fitted with a natural-cubic spline in exposure space and according to AIC scores, the log transformed turbidity data was best fit with 3 df in space of precipitation, log-Clostridium was associated with precipitation using 2 df, whereas a linear association with precipitation was sufficient when analyzing log-concentrations of E. coli and coliforms. Decreased snow cover (Vänersborg) was related to a short-term decrease in raw water quality, most clear regarding turbidity and just about significant regarding coliforms. Including such predictor in the models did not influence effects or lag structure of precipitation, and was not included in final models. Daily mean temperature showed none, or only small effects, on short-term variation and were not included in final models. Turbidity data contained a week day pattern which could be linked to scheduled maintenance of the measuring device (3 times weekly); an average increase of ∼5% per day after filter cleaning was observed, and this effect was adjusted for in all turbidity models.

When comparing estimates between DLNM models and unconstrained distributed lag models (Figure 3) we concluded that the unconstrained designs produced reliable estimates (subject discussed in statistical details (Text S1)), and they were used when analyzing precipitation effect modifications.

## Discussion

### Findings

In this large data study clear relationships between precipitation and decreased river water quality were uncovered. Regression models showed that concentrations of indicator bacteria, but not turbidity, increase exponentially with the amount of observed precipitation (Figure 4, Figure S2), which suggests that prior precipitation in fact predicts fecal contaminations better than mean levels of turbidity at events of heavy rainfall. This conclusion also appears to be valid when creating models with turbidity as a predictor to indicator bacteria; Figure S6 illustrates the association between turbidity and indicator bacteria (lag 0), and for comparison associating indicator bacteria with precipitation two days prior.

When comparing the effect of precipitation across seasons, additional differences between turbidity and indicator bacteria were observed, with a weakened effect on turbidity during summer (Figure 6). This effect decrease on turbidity can only partly be explained by a lower river flow rate in summer periods, which spreads the effects over more lags, and is likely to be a result of seasonal changes in the relation between runoff and rainfall as seasonal soil varies in its ability to absorb precipitation. However, significant precipitation effects on E. coli concentrations were observed across seasons, and we conclude that precipitation is associated with increased risks of introducing pathogens in to river water all year around.

The water quality parameters also showed varying seasonal averages, which to some extent could be explained by prolonged dry or wet weather, but it's likely a result of a combination of factors which might differ with water quality parameter. The inactivation rate of E. coli increases with higher temperatures, but the increased concentrations during winters could also be a consequence of less sunlight intensity [15]. Since E. coli are less long lived in warm temperatures [15], it could also be argued that if water samples were taken closer to the contamination sources a smaller seasonality pattern could have been observed. Additionally, as winter seasons usually holds higher flow rates E. coli survives over longer distances, and higher concentrations are therefore detected downstream. Coliforms showed however less elevated average concentrations during colder periods, and this could be a result of that they are capable of growing in nature and can originate from plant decay. This characteristic is also supported in our data since precipitation explained short-terms variations in coliforms with a larger seasonal variability than other indicator bacteria (Figure S4), and other indicator bacteria may better reflect the extent of fecal pollution. Coliforms were also observed with a declining long-term trend, and a speculative explanation could be that a large paper mill (Wargöns Bruk AB) situated 11 km north of Trollhättan decreased their production during the study period, to finally close in 2008. It is common that pulp and paper mills release high levels of coliforms in their waste [16].

### Further model implications - potentials and limitations

As closures of the river water intake to Alelyckan are determined by concentrations of indicator bacteria (and other causes), the regression models can predict expected closures due to precipitation. Closure of intake due to E. coli counts is protocolled at 400 MPN/100 mL, which the DLNM model predicts to occur two days after precipitation events of 30 mm/24-h. The limit of accepted counts of coliforms are 7000 MPN/100 mL, which DLNM models predict to take place two days after events of 45 mm/24-h, while Clostridium counts of more than 50 CFU/100 mL are predicted to occur two days after events of 39 mm/24-h. These predictions use an average water quality as baseline (i.e. average concentrations are present before the precipitation event) and represent estimates of expected concentrations; peaks in indicator bacteria concentrations can be considerably higher and behave differently than suggested by the estimated lag structure. For example, some model outliers include the maximum observations of E. coli counts (2800 MPN/100 mL and 1400 MPN/100 mL) which were sampled the same day (lag 0) as two of the five heaviest precipitation observations (44.6 and 44.5 mm/24-h). This day had also a turbidity value below the overall mean value, and normal stream flows were registered (500–600 m^3^/s) at these events. DLNM models on coliforms and Clostridium showed also higher effects after events of extreme rainfall when using a more relaxed association with precipitation (3 df), compared to the models presented and suggested by AIC scores. This indicates that the presented estimated effects of precipitation on levels of Clostridium and coliforms are actually conservative at extreme events. Models on E. coli were stable independently of the flexibility parameter, and E. coli is also argued to be the best indicator of bacteriological quality of water [17].

### Related Studies and Perspectives

The findings in this study cannot be directly generalized to other fresh-water supplies because lag structures and effect of rainfall cannot be assumed to be similar. Therefore, assessments of how rainfall increases the risk of highly polluted raw water in other locations should be performed separately. Precipitation data are often well documented and easily accessible which means that water supply producers, once they understand the associations between precipitation and water quality, can better validate the risks, in magnitude and time. To our knowledge, no studies have addressed effects and lag structures of raw water quality with similar statistical methods and precision before, limiting comparison with other studies. Signor et al. [1] addressed variations in water quality (physical, chemical and bacteriological parameters) in an uncontrolled Australian river, where no point sources of contaminations were known. Mean concentrations were compared at runoff events with base flows, with bacteria and parasites counts increasing during increased flow. Kistemann et al. [2] studied three surface water reservoirs in Germany, comparing mean levels before and after runoffs, and detected elevated parasites and indicator bacteria concentrations with increasing water levels. Åström et al. [3] also studied Göta Älv with data from 2004 and found correlations between accumulated rainfall and elevated concentrations of E. coli, and also other pathogen indicators such as intestinal enterococci. Studies have also shown associations between precipitation and outbreaks of gastrointestinal illnesses (GI), indicating that rainfall has an important role in pathogen contamination in water supplies [4]–[7]. Other studies tried to assess the relation between water quality and gastrointestinal illnesses under non-outbreak situations, most commonly using turbidity as exposure variable [18]–[23]. This study suggests that precipitation can be an alternative exposure variable in such studies, since precipitation may better reflects peaks of fecal contamination than turbidity. Studies of rainfall and incidence of GI illnesses under non-outbreak situations are few in number. A study from Milwaukee (WI) reported an increase in emergency department visits 4 days after any amount of rainfall [24]. Associations between heavy rainfall and daily number of nurse advice calls relating to GI problems within the City of Gothenburg has recently been reported [25], and this study supports the causal pathway between rainfall and increased risks due to poor drinking water.

Climate change projections indicate annual precipitation will increase, with more heavy rainfall events in Sweden. Therefore, Göta Älv is expected to encounter more days in the future with inadequate raw water quality than today. Although the drinking water utility Alelyckan has the opportunity to close the water intake, this creates challenges because water in the reservoir lake cannot support the population in Gothenburg with drinking water over long time periods; river water is the only available source of freshwater in the quantities required. Elimination of combined storm and sewage water systems or other systems that result in emergency releases of untreated sewage water into the river should be an important step to increasing river water quality and lowering impacts of heavy precipitation, and to be more prepared for a future climate.

## Supporting Information

Figure S1 **Data–seasonality patterns.** Observations from 2004–2010 plotted within season. Averages projected with a cyclic spline function (9 df). Top row shows observations of river water quality parameters measured near Alelyckan (Gothenburg). A: daily mean turbidity, B: E. coli, C: coliforms and F: Clostridium. Bottom row from left: E: maximum precipitation observation from the three weather stations, F: consecutive wet and dry (negative) days where a wet day was defined as any observed precipitation in any station, G: daily mean temperature observed in Gothenburg and H: stream flow measured in Lilla Edet.
(TIFF)

Figure S2 **Precipitation effects.** Relative effect of daily precipitation (0–54 mm) along 0–15 lags on raw water quality. A: turbidity, B: E. Coli, C: coliforms, D: Clostridium perfringens.
(TIFF)

Figure S3 **Seasonal effect modifications.** Relative effect of daily precipitation (>15 mm/24-h) along 0–8 lags on raw water quality across seasons. A: turbidity, B: E. Coli, C: coliforms, D: Clostridium perfringens.
(TIF)

Figure S4 **R^2^-seasonality patterns.** Variation explained (R^2^-values) across seasons by non-linear precipitation predictors 0–8 day prior observations of river water parameters A: turbidity, B: E. Coli, C: coliforms, D: Clostridium perfringens. Colors represent the three different precipitation stations and horizontal dotted lines represent the average R^2^.
(TIFF)

Figure S5 **Stream flow effect modifications.** Estimated relative effect on turbidity of a rainfall event of >15 mm along 0–15 lags days at different stream flows (quartiles). Vertical bars represent 95% CI. Vertical lines represent estimated effect peaks.
(TIFF)

Figure S6 **Turbidity, precipitation and indicator bacteria.** Associations (penalized splines, max 5 df) between turbidity and indicator bacteria (lag 0) and precipitation and indicator bacteria (lag 2) (A and B: E. coli, C and D: coliforms, E and F: Clostridium), together with model residuals (dots). Blue dotted lines represent mean levels, and red dotted lines represent unaccepted levels for open raw water intake at Alelyckan drinking water utility.
(TIFF)

Table S1 **Monthly statistics of observations in river water during 2004–2010.**
(DOCX)

Text S1 **Statistical details.**
(DOCX)

## References

[1] Signor RS, Roser DJ, Ashbolt NJ, Ball JE (2005) Quantifying the impact of runoff events on microbiological contaminant concentrations entering surface drinking source waters. J Water Health 3: 453–468. 16459849 10.2166/wh.2005.052

[2] Kistemann T, Classen T, Koch C, Dangendorf F, Fischeder R, et al (2002) Microbial load of drinking water reservoir tributaries during extreme rainfall and runoff. Appl Environ Microbiol 68: 2188–2197. 11976088 10.1128/AEM.68.5.2188-2197.2002 PMC127524

[3] Astrom J, Petterson S, Bergstedt O, Pettersson TJR, Stenstrom TA (2007) Evaluation of the microbial risk reduction due to selective closure of the raw water intake before drinking water treatment. Journal of Water and Health 5: 81–97. 17890838 10.2166/wh.2007.139

[4] Curriero FC, Patz JA, Rose JB, Lele S (2001) The association between extreme precipitation and waterborne disease outbreaks in the United States, 1948–1994. Am J Public Health 91: 1194–1199. 11499103 10.2105/ajph.91.8.1194 PMC1446745

[5] Nichols G, Lane C, Asgari N, Verlander NQ, Charlett A (2009) Rainfall and outbreaks of drinking water related disease and in England and Wales. J Water Health 7: 1–8. 18957770 10.2166/wh.2009.143

[6] Thomas KM, Charron DF, Waltner-Toews D, Schuster C, Maarouf AR, et al (2006) A role of high impact weather events in waterborne disease outbreaks in Canada, 1975–2001. Int J Environ Health Res 16: 167–180. 16611562 10.1080/09603120600641326

[7] Cann KF, Thomas DR, Salmon RL, Wyn-Jones AP, Kay D (2013) Extreme water-related weather events and waterborne disease. Epidemiol Infect 141: 671–686. 22877498 10.1017/S0950268812001653 PMC3594835

[8] Reynolds KA, Mena KD, Gerba CP (2008) Risk of waterborne illness via drinking water in the United States. Rev Environ Contam Toxicol 192: 117–158. 18020305 10.1007/978-0-387-71724-1_4 PMC7120101

[9] Delpla I, Jung AV, Baures E, Clement M, Thomas O (2009) Impacts of climate change on surface water quality in relation to drinking water production. Environ Int 35: 1225–1233. 19640587 10.1016/j.envint.2009.07.001

[10] Swedish Commission on Climate and Vulnerability (2007) Sweden facing climate change – threats and opportunities, Stockholm. Swedish Governmental Official Reports (SOU) 2007:60. Final report. Available: http://www.sweden.gov.se/sb/d/8704/a/89334 Sverige inför klimatförandringarna. Bilaga B 13: Dricksvattenförsörjning i förändrat klimat.

[11] Wood SN (2011) Fast stable restricted maximum likelihood and marginal likelihood estimation of semiparametric generalized linear models. Journal of the Royal Statistical Society Series B-Statistical Methodology 73: 3–36.

[12] Gasparrini A, Armstrong B, Kenward MG (2010) Distributed lag non-linear models. Stat Med 29: 2224–2234. 20812303 10.1002/sim.3940 PMC2998707

[13] Team R (2010) R: A Language and Environment for Statistical Computing. R Foundation for Statistical Computing, Vienna, Austria, 2007. ISBN 3-900051-07-0.

[14] Gasparrini A (2011) Distributed lag linear and non-linear models in R: the package dlnm. Journal of Statistical Software 43(8): 1–20. PMC3191524 22003319

[15] Blaustein RA, Pachepsky Y, Hill RL, Shelton DR, Whelan G (2013) Escherichia coli survival in waters: temperature dependence. Water Res 47: 569–578. 23182082 10.1016/j.watres.2012.10.027

[16] Gauthier F, Archibald F (2001) The ecology of “fecal indicator” bacteria commonly found in pulp and paper mill water systems. Water Research 35: 2207–2218. 11358300 10.1016/s0043-1354(00)00506-6

[17] Odonkor ST, Ampofo JK (2013) Escherichia coli as an indicator of bacteriological quality of water: an overview. Microbiology Research 4: e2.

[18] Egorov AI, Naumova EN, Tereschenko AA, Kislitsin VA, Ford TE (2003) Daily variations in effluent water turbidity and diarrhoeal illness in a Russian city. Int J Environ Health Res 13: 81–94. 12745350 10.1080/0960312021000071567

[19] Gilbert ML, Levallois P, Rodriguez MJ (2006) Use of a health information telephone line, Info-sante CLSC, for the surveillance of waterborne gastroenteritis. J Water Health 4: 225–232. 16813015

[20] Morris R, Naumova EN, Levin R, Munasinghe R (1996) Temporal variation in drinking water turbidity and diagnosed gastroenteritis in Milwaukee. American journal of public health 86: 237–239. 8633742 10.2105/ajph.86.2.237 PMC1380334

[21] Schwartz J, Levin R, Goldstein R (2000) Drinking water turbidity and gastrointestinal illness in the elderly of Philadelphia. J Epidemiol Community Health 54: 45–51. 10692962 10.1136/jech.54.1.45 PMC1731533

[22] Schwartz J, Levin R, Hodge K (1997) Drinking water turbidity and pediatric hospital use for gastrointestinal illness in Philadelphia. Epidemiology 8: 615–620. 9345659 10.1097/00001648-199710000-00001

[23] Tinker SC, Moe CL, Klein M, Flanders WD, Uber J, et al (2010) Drinking water turbidity and emergency department visits for gastrointestinal illness in Atlanta, 1993–2004. Journal of Exposure Science and Environmental Epidemiology 20: 19–28. 18941478 10.1038/jes.2008.68 PMC3752848

[24] Drayna P, McLellan SL, Simpson P, Li SH, Gorelick MH (2010) Association between Rainfall and Pediatric Emergency Department Visits for Acute Gastrointestinal Illness. Environmental Health Perspectives 118: 1439–1443. 20515725 10.1289/ehp.0901671 PMC2957926

[25] Tornevi A, Axelsson G, Forsberg B (2013) Association between precipitation upstream of a drinking water utility and nurse advice calls relating to acute gastrointestinal illnesses. PLoS One 8: e69918. 23875009 10.1371/journal.pone.0069918 PMC3713056

