# Supplementary figures and images for: Precipitation Effects on Microbial Pollution in a River: Lag Structures and Seasonal Effect Modification
Source: PLoS One. 2014 May 29;9(5):e98546. doi: 10.1371/journal.pone.0098546 (PMC4038599; doi:10.1371/journal.pone.0098546)

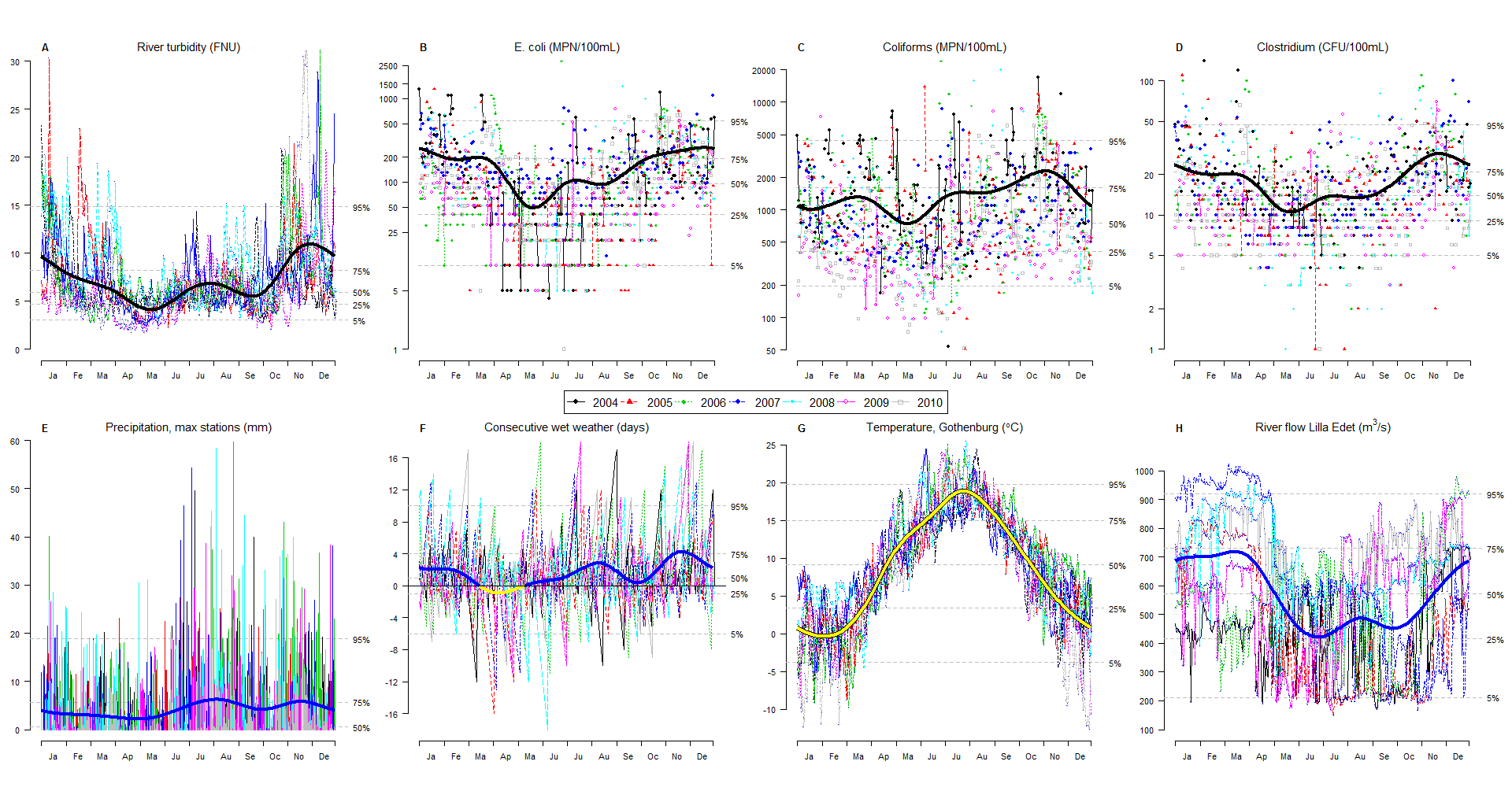

Supplement: Figure S1 — Data–seasonality patterns. Observations from 2004–2010 plotted within season. Averages projected with a cyclic spline function (9 df). Top row shows observations of river water quality parameters measured near Alelyckan (Gothenburg). A: daily mean turbidity, B: E. coli, C: coliforms and F: Clostridium. Bottom row from left: E: maximum precipitation observation from the three weather stations, F: consecutive wet and dry (negative) days where a wet day was defined as any observed precipitation in any station, G: daily mean temperature observed in Gothenburg and H: stream flow measured in Lilla Edet. (TIFF) [file pone.0098546.s001.tif]

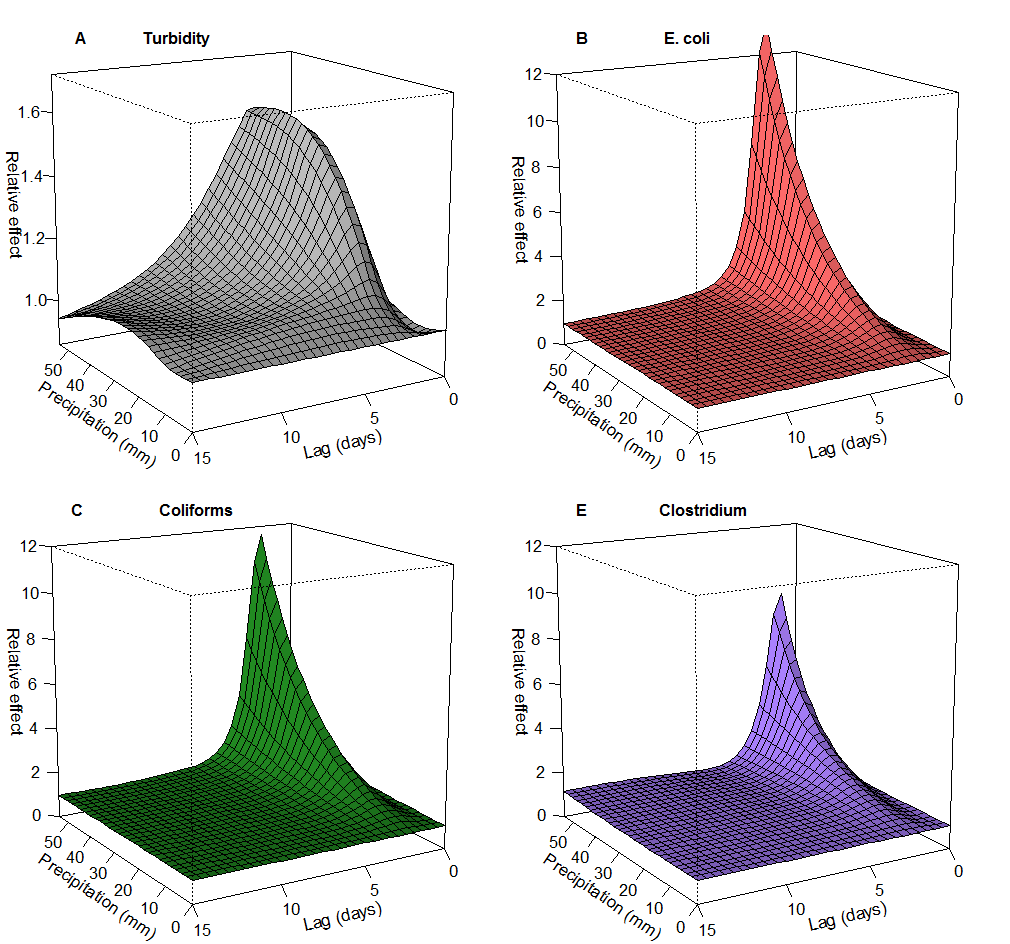

Supplement: Figure S2 — Precipitation effects. Relative effect of daily precipitation (0–54 mm) along 0–15 lags on raw water quality. A: turbidity, B: E. Coli, C: coliforms, D: Clostridium perfringens. (TIFF) [file pone.0098546.s002.tif]

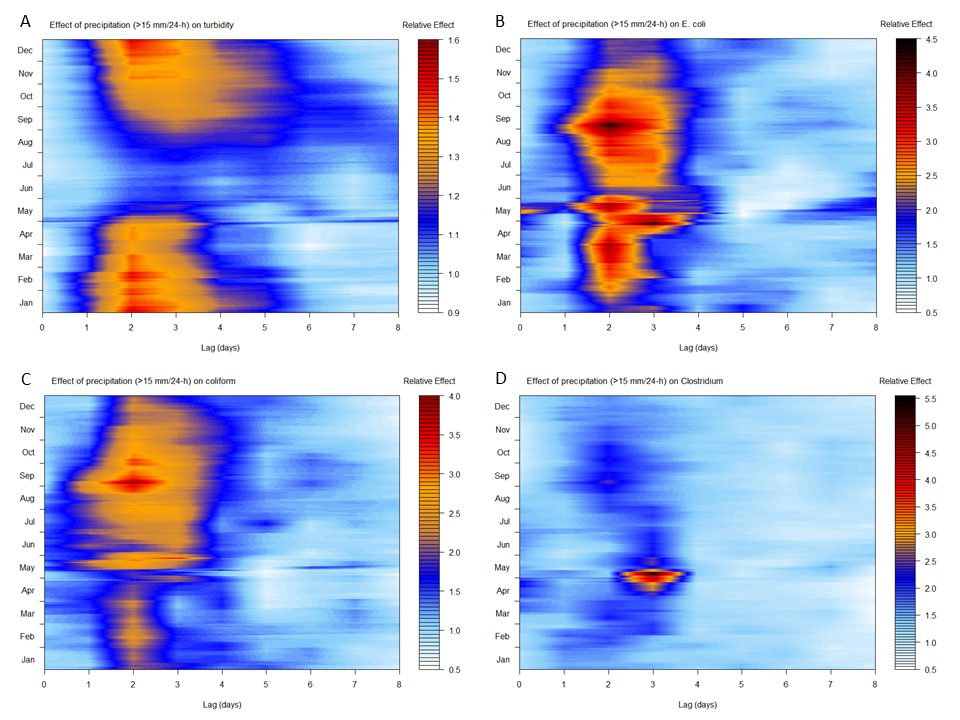

Supplement: Figure S3 — Seasonal effect modifications. Relative effect of daily precipitation (>15 mm/24-h) along 0–8 lags on raw water quality across seasons. A: turbidity, B: E. Coli, C: coliforms, D: Clostridium perfringens. (TIF) [file pone.0098546.s003.tif]

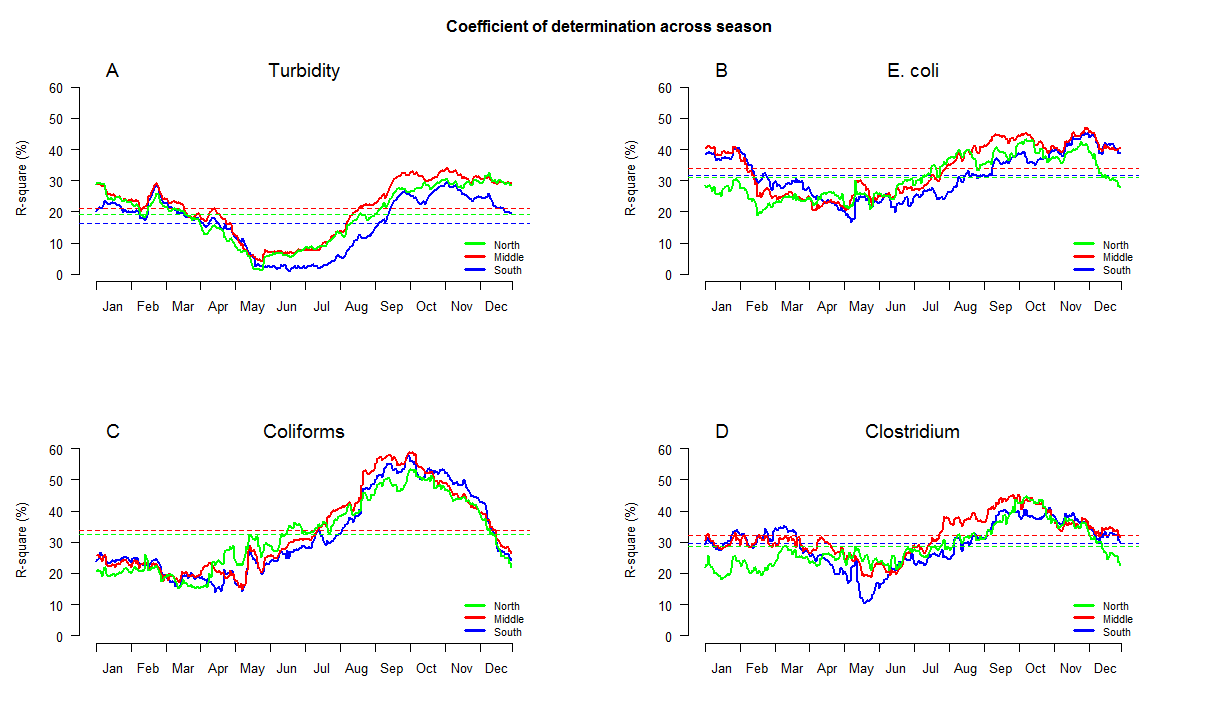

Supplement: Figure S4 — R2-seasonality patterns. Variation explained (R2-values) across seasons by non-linear precipitation predictors 0–8 day prior observations of river water parameters A: turbidity, B: E. Coli, C: coliforms, D: Clostridium perfringens. Colors represent the three different precipitation stations and horizontal dotted lines represent the average R2. (TIFF) [file pone.0098546.s004.tif]

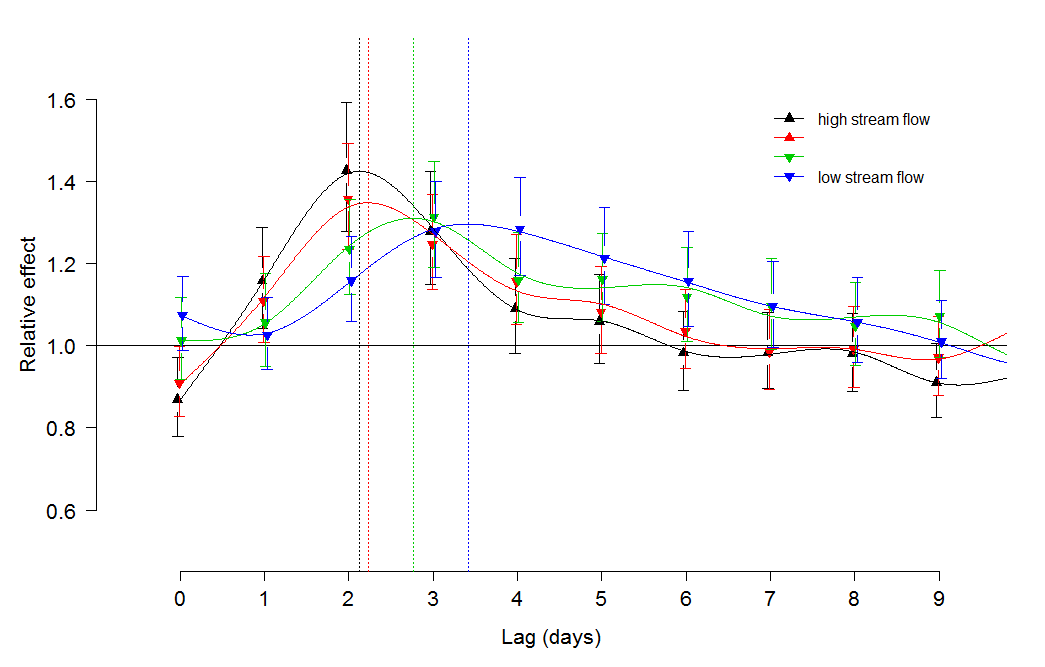

Supplement: Figure S5 — Stream flow effect modifications. Estimated relative effect on turbidity of a rainfall event of >15 mm along 0–15 lags days at different stream flows (quartiles). Vertical bars represent 95% CI. Vertical lines represent estimated effect peaks. (TIFF) [file pone.0098546.s005.tif]

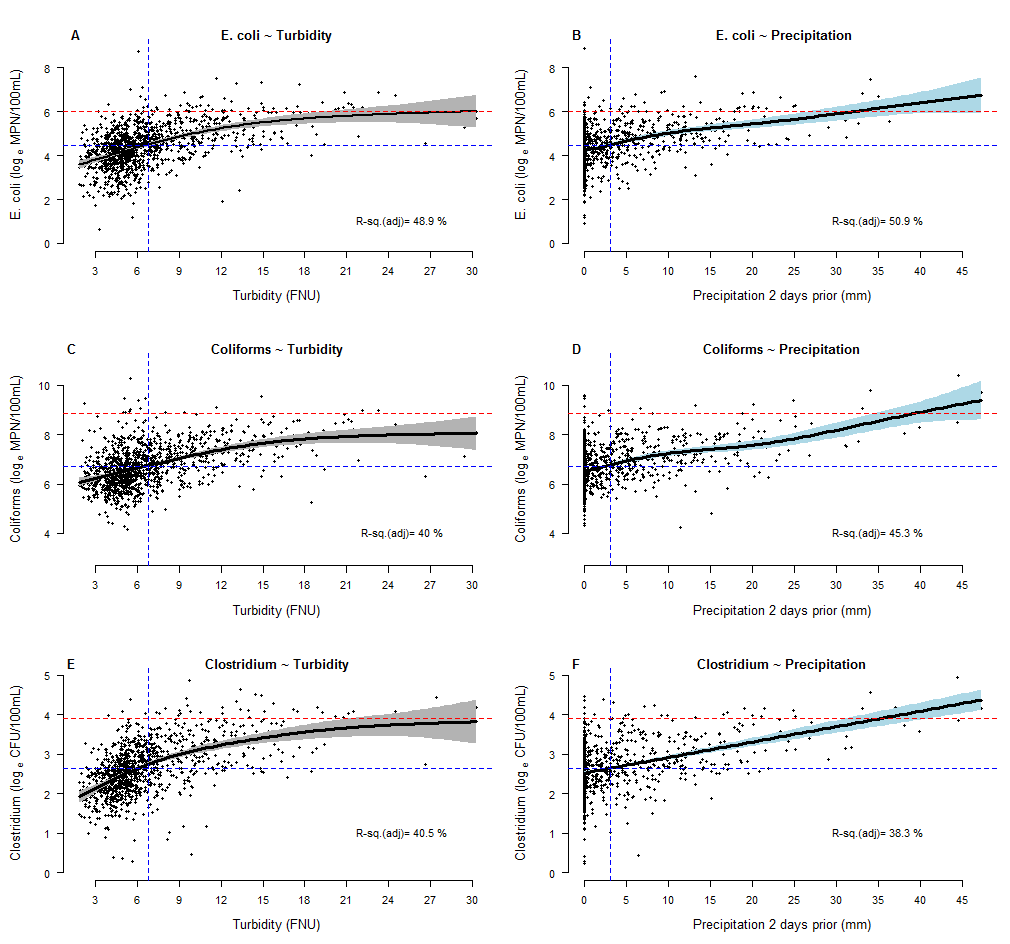

Supplement: Figure S6 — Turbidity, precipitation and indicator bacteria. Associations (penalized splines, max 5 df) between turbidity and indicator bacteria (lag 0) and precipitation and indicator bacteria (lag 2) (A and B: E. coli, C and D: coliforms, E and F: Clostridium), together with model residuals (dots). Blue dotted lines represent mean levels, and red dotted lines represent unaccepted levels for open raw water intake at Alelyckan drinking water utility. (TIFF) [file pone.0098546.s006.tif]
