# Supplementary material for: Precipitation Effects on Microbial Pollution in a River: Lag Structures and Seasonal Effect Modification
Source: PLoS One. 2014 May 29;9(5):e98546. doi: 10.1371/journal.pone.0098546 (PMC4038599; doi:10.1371/journal.pone.0098546)
Supplement: Table S1 — Monthly statistics of observations in river water during 2004–2010. (DOCX) [file pone.0098546.s007.docx]

|  | Month | N | Missing | Mean | St.Dev | Minimum | 25%:tile | Median | 75%:tile | Maximum |
| --- | --- | --- | --- | --- | --- | --- | --- | --- | --- | --- |
| Turbidity (FNU) | Jan | 217 | 0 | 9.9 | 4.3 | 3.6 | 6.5 | 9.2 | 12.7 | 30.3 |
|  | Feb | 198 | 0 | 7.1 | 3.8 | 3.1 | 4.7 | 5.8 | 7.7 | 23.0 |
|  | Mar | 217 | 0 | 6.9 | 3.3 | 2.1 | 4.5 | 6.0 | 8.8 | 19.3 |
|  | Apr | 208 | 2 | 4.9 | 2.4 | 1.7 | 3.1 | 4.6 | 5.7 | 17.4 |
|  | May | 216 | 1 | 4.2 | 1.4 | 1.6 | 3.1 | 4.2 | 5.1 | 9.7 |
|  | Jun | 209 | 1 | 5.6 | 1.5 | 2.2 | 4.8 | 5.4 | 6.3 | 11.9 |
|  | Jul | 216 | 1 | 6.3 | 1.9 | 3.4 | 5.0 | 5.8 | 7.2 | 14.3 |
|  | Aug | 217 | 0 | 6.6 | 2.0 | 3.7 | 5.3 | 6.1 | 7.6 | 15.2 |
|  | Sep | 210 | 0 | 5.9 | 2.3 | 2.2 | 4.3 | 5.3 | 6.9 | 14.9 |
|  | Oct | 217 | 0 | 7.0 | 3.8 | 2.0 | 4.4 | 5.6 | 8.5 | 20.9 |
|  | Nov | 210 | 0 | 10.7 | 5.6 | 2.3 | 6.9 | 9.3 | 12.0 | 33.7 |
|  | Dec | 215 | 2 | 9.2 | 4.7 | 3.2 | 6.0 | 7.9 | 11.1 | 31.7 |
|  |  |  |  |  |  |  |  |  |  |  |
| E. coli (MPN/100mL) | Jan | 99 | 118 | 263.6 | 243.8 | 20.0 | 97 | 190 | 350 | 1300 |
|  | Feb | 88 | 110 | 186.1 | 206.4 | 10 | 63 | 120 | 200 | 1100 |
|  | Mar | 97 | 120 | 180 | 228.9 | 5 | 52 | 90 | 190 | 1100 |
|  | Apr | 95 | 115 | 128.2 | 160.8 | 5 | 31 | 86 | 160 | 1000 |
|  | May | 98 | 119 | 53.8 | 50.1 | 5 | 20 | 41 | 73 | 270 |
|  | Jun | 95 | 115 | 92.1 | 297.8 | 1 | 10 | 31 | 74 | 2800 |
|  | Jul | 97 | 120 | 74.3 | 124.2 | 5 | 10 | 31 | 63 | 710 |
|  | Aug | 98 | 119 | 110.4 | 122.1 | 5 | 41 | 73 | 132.5 | 760 |
|  | Sep | 99 | 111 | 140.9 | 175.2 | 5 | 41 | 85 | 190 | 1400 |
|  | Oct | 102 | 115 | 203.7 | 217.8 | 10 | 63 | 125 | 240 | 1200 |
|  | Nov | 95 | 115 | 246.5 | 164.5 | 23 | 120 | 200 | 330 | 790 |
|  | Dec | 93 | 124 | 196 | 170 | 10 | 90 | 140 | 230 | 1100 |
|  |  |  |  |  |  |  |  |  |  |  |
| Coliforms (MPN/100mL) | Jan | 99 | 118 | 1328 | 1186 | 160 | 550 | 910 | 1700 | 7300 |
|  | Feb | 88 | 110 | 1114 | 1105 | 160 | 460 | 695 | 1400 | 5500 |
|  | Mar | 97 | 120 | 1204 | 1064 | 98 | 400 | 910 | 1600 | 4900 |
|  | Apr | 95 | 115 | 1139 | 1406 | 98 | 360 | 560 | 1500 | 8200 |
|  | May | 98 | 119 | 717.9 | 770.2 | 74 | 272.5 | 460 | 880 | 5500 |
|  | Jun | 95 | 115 | 1133 | 3065 | 74 | 210 | 380 | 760 | 24000 |
|  | Jul | 97 | 120 | 1171 | 1791 | 51 | 300 | 490 | 1500 | 12000 |
|  | Aug | 98 | 119 | 1674 | 2138 | 240 | 570 | 1050 | 1900 | 16000 |
|  | Sep | 99 | 111 | 1504 | 2362 | 170 | 450 | 780 | 1600 | 20000 |
|  | Oct | 102 | 115 | 2351 | 2806 | 240 | 558 | 1300 | 2900 | 17000 |
|  | Nov | 95 | 115 | 2009 | 1737 | 230 | 910 | 1500 | 2600 | 12000 |
|  | Dec | 93 | 124 | 1061.6 | 900.5 | 170 | 435 | 750 | 1450 | 4100 |
|  |  |  |  |  |  |  |  |  |  |  |
| Clostridium (CFU/100mL) | Jan | 90 | 127 | 24.8 | 19.1 | 4 | 12 | 19.5 | 31.3 | 110 |
|  | Feb | 80 | 118 | 20 | 18.9 | 5 | 9 | 14 | 22.8 | 140 |
|  | Mar | 94 | 123 | 21 | 20.6 | 5 | 10 | 13 | 23.3 | 120 |
|  | Apr | 88 | 122 | 14 | 11.4 | 3 | 7 | 11 | 16 | 83 |
|  | May | 90 | 127 | 12.1 | 7 | 1 | 8 | 10.5 | 15 | 41 |
|  | Jun | 90 | 120 | 11.8 | 7.5 | 1 | 7 | 10 | 14.3 | 43 |
|  | Jul | 84 | 133 | 13.1 | 10.3 | 1 | 7 | 10 | 15 | 56 |
|  | Aug | 86 | 131 | 14 | 7.8 | 2 | 8 | 12 | 19 | 38 |
|  | Sep | 84 | 126 | 15.7 | 13 | 2 | 8 | 12 | 21 | 81 |
|  | Oct | 90 | 127 | 21.1 | 16.4 | 3 | 10 | 17.5 | 24.3 | 89 |
|  | Nov | 87 | 123 | 28.9 | 17.5 | 2 | 18 | 25 | 38 | 110 |
|  | Dec | 91 | 126 | 22.1 | 16.2 | 4 | 12 | 17 | 27 | 100 |
