# Supplementary material for: Precipitation Effects on Microbial Pollution in a River: Lag Structures and Seasonal Effect Modification
Source: PLoS One. 2014 May 29;9(5):e98546. doi: 10.1371/journal.pone.0098546 (PMC4038599; doi:10.1371/journal.pone.0098546)
Supplement: Text S1 — Statistical details. (DOCX) [file pone.0098546.s008.docx]

**Precipitation effects on microbial pollution in a river: lag structures and seasonal effect modification – Supplementary material**

**Statistical details**

This supplementary material describes details of the generalized additive regression models (GAM) used to assess effects of precipitation on raw water quality parameters. All raw water parameters were assumed to follow a log-normal distribution, and data were log-transformed with the natural logarithm. The statistical software R (v 2.15.2) [13], together with MGCV [11] and DLNM [14] packages were used. References can be found in the main article.

**Main effects of precipitation**

To assess short-term effects of precipitation we created time series regression models as:

$\begin{matrix} E(\log(y_{t}))=\alpha+s(time_{t}|df)_{t}+f_{1}(precipitation_{t:(t-L)}) \\ +f_{2}(temperature_{t:(t-L)})+f_{3}(snow depth_{t:(t-L)})+ f_{4}(DOW_{t}) \end{matrix}$ (1)

, where the expected value (*E*) of the raw water quality parameter (*y*) at day *t*, was determined by functions describing seasonal and long-term trends (function$s$) and short-term variations (functions$f$).

**Long-term trends**. Long-term variations of the outcome was described with a (thin-plate) spline function projecting a moving average by letting the variable *time* represents an ordered discrete count throughout all observation days. This simultaneously decomposes seasonal patterns and long-term time trends. The elasticity in *s* is defined by degrees of freedom (df), and was set differently with different outcomes: 7 df / year modeling indicator bacteria and 10 df / year modeling turbidity.

**Distributed lags**. The effect of precipitation on the outcome was analyzed over a lag period (*L*), which was initially set to 0-15 days. Thus, function$f_{1}$ includes daily precipitation observations *L* days prior *t*. As serial correlation between observations can be present in time series data, multicollinearity problems can arise, and therefore constrained distributed lag model designs are preferably applied to analyze distribution of delayed associations. Distributed Lag Non-linear Models (DLNM) [12], a statistical package within the software R [13] allow for such designs, where the lag space and exposure space is simultaneously associated to the outcome with a cross-basis function. A simpler design, and easier to use when stratifying the analysis on irregular time segments, is to fit the lagged predictors as independent variables (an unconstrained distributed lag design), but then serial correlations could thus lead to unreliable estimates. To validate the reliability of using unconstrained distributed lag models on this data we compared their estimated precipitation effects in lag space with precipitation effects produced by DLNM models with natural cubic spline function describing the distribution in lag space.

**Precipitation**. To achieve an overall effect estimate of heavy precipitation, we factorized precipitation observations to {0 mm, 0-15 mm, >15 mm}, where 15 mm is approximately the regional 95 percentile. In DLNM models we also examined continuous non-linear associations between precipitation and raw water quality with natural cubic splines, and let AIC scores decide the best parameter setting (degree of non-linearity).

Precipitation observations at the three meteorological stations were highly correlated and their effect on raw water parameters were therefore analyzed and compared in separate models. The predictive ability of observations from each weather station was evaluated by calculating their ability to explain variances of the outcomes (coefficient of determination: R^2^).

**Consecutive weather**. Distributed lag models don’t consider effects of consecutive events. Therefore we also analyzed effects of consecutive days of dry and wet weather, defining a wet weather day as a day with any precipitation (>0 mm). Thus, a variable was created counting consecutive wet days (positive numbers) and dry days (negative numbers). We considered this variable as continuous predictor to raw water parameters, allowing for non-linear associations with a penalized spline, and alternately as a factorial predictor that was truncated with the restriction to hold at least 30 observations in each level. Using precipitation observations from the middle station the factor variable were then truncated at 11 consecutive dry days and 7 consecutive wet days.

**Short-term effects**. Thus, the function $f_{1}$ in (1) represent the associations with precipitation (factorized or continuous variable), either with a cross-basis function (DLNM), or as an unconstrained distributed lag design (with a separate association for each lag), or alternatively a relationship with consecutive dry or wet days.

Additionally, we analyzed if daily mean temperature and change in upstream snow depth using DLNM designs ($f_{2}$ $f_{3}$ in (1)), and day of week (*DOW in (1)*), were associated with short-term variations in raw water quality data. If these predictors influenced estimated precipitation effects, or importantly improved model diagnostics they were included in the final model.

**Precipitation effect modifications**

As the study area has distinct seasonality in temperature, daylight, ecology etc., we examined how short term effects of precipitations on raw water quality parameters vary with season. We also investigated precipitation effects at different stream flows to examine possible differences in size and lag structure. Information from the analysis of main effects (model 1) (covariates and sufficient length of lag structure together with choice of distributed lag design) was brought forward to these analyses.

**Two-stage analysis.** The analysis was performed by a two-stage model approach by first adjusting for long-term trends (and week day patterns) using the complete data series, then precipitation effects were evaluated by analyzing the decomposed data (the residuals) within specific time periods. Hence, in the first stage we fit raw water quality data with the model:

$E(\log(y_{t}))=\alpha+s(time_{t}|df)+ DOW_{t}$ (2)

, where the notations and parameter setting (df) were the same as in model (1). The difference between the observed concentrations and the modeled expected concentrations from (2) was calculated: , and becomes a seasonally and long-term trend adjusted raw quality parameter at day *t*. The weekday factor (*DOW*) was only included in (2) when analyzing turbidity.

In the second stage we evaluated the effect of precipitation (factorized to: {0 mm, 0-15 mm, >15 mm}) with an unconstrained distributed lag regression model as

(3)

, thus we restrict the model to cover 0-8 days of precipitation prior *t,* and stratified the regression analysis to time periods as follows.

**Stratifying by season.** We considered effect modifications by season by including 90-day per year periods in model (3). Thus, the analyzed observations included recurrent series of 90 days from 365 days periods. By changing the inclusion period, for example with a step of one day, and repeating the analysis, a seasonal moving average of effects could be assessed. To clarify, in the first model day 1-90 every year was included, the second model included day 2-91 every year, and so forth until model (3) was repeated 364 times.

Assessment of predictive ability of precipitation through seasons where made by extracting R^2^ values from each model, and we compared them using precipitation data from the three different metrological stations. For this purpose we fitted continuous non-linear associations with precipitation using penalized thin-plate spline functions (max 4 df).

**Stratifying by stream flow.** To assess precipitation effects at different stream flows we designated the adjusted observations () into time segments separated by flow quartiles. We studied the effect of precipitation in the four groups in separate models with turbidity as outcome, and to better evaluate and compare lag peaks effects we fitted a smooth spline through the estimated coefficients describing the lag distribution.
